# Short Communication: Rotavirus Group A Occurrence in Rural Water Source Samples in a Midwest Region State of Brazil, Comparing Wet and Dry Seasons

**DOI:** 10.3390/v16091452

**Published:** 2024-09-12

**Authors:** Graziela Picciola Bordoni, Lucas Candido Gonçalves Barbosa, Fernando Santos Lima, Mônica de Oliveira Santos, José Daniel Gonçalves Vieira, Thais Reis Oliveira, Paulo Sérgio Scalize, Lilian Carla Carneiro

**Affiliations:** 1 Institute of Tropical Pathology and Public Health, Federal University of Goiás, 235 Street, Goiânia 74605-050, Brazil; 2 Medicine College, Federal University of Goiás, 235 Street, Goiânia 74690-900, Brazil; 3 School of Civil Engineering, Federal University of Goiás, Q University Street, lt. 1488, bl. A, sl. 7, Goiânia 74605-010, Brazil

**Keywords:** rotavirus, water contamination, dry period, rainy period

## Abstract

Identified as a potential reference pathogen by the WHO Guidelines for Drinking-Water Quality, Rotavirus (RV) is among the main enteric viruses that cause waterborne diseases. The aim of this study was to identify and correlate the presence of RV in collective and individual water sources of rural communities in the state of Goiás, within the seasons in which the collections were made (rainy and dry seasons). For this, 86 water samples in the dry period and 160 samples in the rainy period were collected. Concentration of water samples, extraction of viral genetic material and molecular tests were performed. When analyzing the presence of RV in the samples, taking into consideration the period studied, RV was found to be more prevalent in the dry season (54.7%) than in the rainy season (20%), showing a strong statistical association with the dry season (*p*-value < 0.001). The presence of pathogenic microorganisms in water is a public risk issue, enabling the emergence of outbreaks, endemics and epidemics. In the present research, there was an association between the presence of Rotavirus and the dry period of the year when compared to the rainy period.

## 1. Introduction

Water contaminated by microbial pathogens poses a considerable risk to public health in general, as these pathogens are responsible for outbreaks of waterborne diseases and thus lead to a high incidence of morbidity and mortality, especially in underdeveloped countries [1,2,3]. Viruses and bacteria are among the main pathogens that cause waterborne diseases [4,5]. Named enteric viruses, they are microorganisms excreted in the feces of infected individuals and that can contaminate humans through direct person-to-person contact by fomites, droplets and contact with secretions, or are capable of contaminating indirectly through ingestion of contaminated water or food or through recreational activities in aquatic environments [3]. They have been found in both surface and groundwater sources [6]. Enteroviruses, adenoviruses, Noroviruses, Hepatitis A and E viruses, as well as Rotaviruses are among the viruses most associated with human waterborne diseases [6].

Viruses of the genus Rotavirus (RV) are non-enveloped viruses with an icosahedral capsid, belonging to the *Sedoreoviridae* family and measuring from 70 nm to 100 nm in diameter [6,7,8]. They have in their viral genome 11 segments of double-stranded RNA and each of these segments encodes a specific viral protein, six of which are structural, called viral proteins (VPs) and six are non-structural (NSPs) [6,7]. RVs are classified into ten species (A-J) based on their antigenic differences and differences in the sequence of the VP6 capsid protein, with group A (RVA) being the predominant cause of RV gastroenteritis [9,10].

Responsible for being one of the main causes of severe viral gastroenteritis that affects the pediatric population, RV is responsible for a significant portion of deaths related to diarrhea among children under five years old [7,11]. The number of cases of deaths from gastroenteritis with diarrhea, after the introduction of vaccines against RV, managed to be significantly reduced in some countries in Latin America, North America, Europe and Australia, with Brazil being one of the pioneers in the introduction of the RV-A vaccine in its National Immunization Program (PNI) [12]. Hospitalization rates for acute diarrhea reduced by 14% after the introduction of RV vaccination in Brazil, with an average of 115.74 hospitalization cases. After vaccination, hospitalization cases in the year of 2006 decreased to an average of 85.84 cases and in 2007 decreased to 59.94 cases, reducing by 25.8% and 48.0%, respectively [13]. In the United States, hospitalization cases of children under 5 years of age before RV vaccination were 55,000–70,000. Since the introduction of vaccination, according to the Center for Disease Control (CDC) this number has decreased to 40,000 from 50,000 hospitalizations [14].

Therefore, the aim of this study was to identify and correlate the presence of Rotavirus with collective and individual water sources (deep tubular wells, shallow tubular wells, shallow dug wells, springs, surface water, rainwater stored in cisterns and water trucks that were collected from surface water) of rural communities in the state of Goiás, and also to correlate this with the seasons in which the collections were made (rainy and dry season) to assist in research and understanding the seasonality of the virus.

## 2. Materials and Methods

### 2.1. Description of Sample Collection Sites during Dry and Rainy Season

This study was carried out using water samples from seven types of sources (serving as Water Supply Systems (WSSs) and/or Individual Alternative Solutions (IASs) for all water uses (drinking, bathing, washing vegetables, fruits and legumes, cooking and other uses), collected in rural and traditional communities (settlements and quilombola and riverside communities) in municipalities located in the state of Goiás. The water sources were as follows: shallow wells, shallow tubular wells, deep tubular wells, cisterns (rainwater), surface water, spring water and water from tanker trucks.

In the state of Goiás, the rainy season lasts from October to April (summer), while the dry period lasts from May to September (winter). The sample collection in the dry season was carried out in April to October of 2019 and a total of 86 samples were collected, while in the rainy season, 160 samples were collected during November and December of 2021.

The dry season collections were carried out in quilombola communities and settlements located in 16 municipalities, and the rainy season collections were carried out in 22 municipalities that included settlements and quilombola and riverside communities. All data on municipalities and their communities are in Table 1.

A total of 500 mL of water was collected in sterile bottles for viral concentration and quantification. The samples were transported immediately to the laboratory after collection, always kept in thermal boxes. In the laboratory, they were kept at 4 °C until further analysis.

### 2.2. RV Concentration

The adsorption–elution methodology was chosen for the concentration of the water samples, collected both in the dry and rainy seasons. The technique was described by Katayama et al. (2002) [15] and was performed with modifications, similar to what was carried out by Vecchia et al. (2012) [16].

### 2.3. The Extraction of Viral Nucleic Acids of RV in the Samples

The Rotavirus genetic material was extracted using a Mini Spin Virus DNA/RNA (KASVI^®^) extraction kit, following the manufacturer’s instructions. At the end of the reaction, the kit provided 30 μL of genetic material eluted in nuclease-free water, which was stored at −80 °C until the reverse transcriptase qPCR (RT-qPCR) step.

### 2.4. RV Positive Control

The positive control was chosen and aligned through the NCBI Database (Gen-Bank), with accession number HM348746 (human Rotavirus A strain mani-265/07 of the structural protein VP6 gene) and was then synthesized by the company Molecular Biotechnology LTDA^®^.

The number of viral genomic copies of the RV-A standard control was obtained from its RNA mass, quantified in 1 μL using the Nano Vue TM Plus^®^ spectrophotometer (GE Healthcare Life Sciences), resulting in a concentration of 37 ng/µL. Subsequently, this mass was converted into the number of RNA molecules. For these calculations, it was assumed that the human Rotavirus VP6 structural protein genome contained 1266 base pairs. For the RV control, it was also considered that all genomic RNA was converted into cDNA. For the quantification of viral particles, five-point standard curves were made in serial dilutions at factor 10 of the Rotavirus Standard Control, from a dilution of 10^−1^ to 10^−5^, starting from 5.6 × 10^8^ genomic copies per reaction. The serial dilution of the positive control showed the following titrations: 10^−1^ with 5.6 × 10^8^ genomic copies, for 10^−2^ with 5.6 × 10^8^, for 10^−3^ with 5.6 × 10^7^, for 10^−4^ with 5.6 × 10^6^ and for 10^−5^ with 5.6 × 10^5^.

### 2.5. Molecular Analysis: The Detection and Quantification of RV in the Samples

A Sybr Green^®^ (double-stranded DNA intercalator) was used as the dye for the detection and quantification of the RV in the samples. An RT-qPCR single step was performed using a Light Cycler^®^ 480 Real-Time PCR System (Roche Molecular Systems, Inc., Pleasanton, CA, USA) with 96-well plates and analyzed using Light Cycler^®^ 480 software, version 1.5. The RT-qPCR results were given in genomic copies per liter (GC/L). Primers for RV were specific for RV group A. The primer used is described in Table 2.

### 2.6. RT-qPCR

The protocol followed for the RT-qPCR reaction is described as follows: 7.5 μL of Real Time PCR master mix solution—Sybr Green/ROX 2x (QuatroG^®^), 0.5 μL of each primer (10 pmol/μL), 0.5 μL of the reverse transcriptase enzyme, provided by researchers from the Institute of Molecular Biology of Paraná (IMBP), Viviane Monteiro Goes, Mariely Cordeiro Estrela and Priscila Zanette de Souza, 5 μL of the sample and 1 μL of DNAse/RNAse-free ultrapure water, adding a final volume of 15 μL. The cycles performed in the thermocycler consisted of only 1 cycle of 15 min at 45 °C and 1 cycle of 2 min at 95 °C to activate the RT enzyme and DNA polymerase, respectively. After this initial step, 40 cycles were followed, consisting of a 15 s step at 95 °C for strand denaturation and a 1 min step at 60 °C for annealing the oligonucleotides to the strand and extension. In each round of qPCR, a negative sample (NTC—in the template control) was used, which contained DNAse/RNAse free water in place of the sample.

### 2.7. Statistical Analysis

The sample calculation was carried out to guarantee the accuracy of the results, and the comparison between seasonal periods considered results from research carried out in dry and rainy periods, with an emphasis on research by Steyer and collaborators (2011) [17] and Castells and collaborators (2018) [18]. As a result, the beta power (β) resulted in 97%, considering an alpha (α) of 5%. Based on the imputed data, the sample size was 86 in the dry period and 160 in the rainy period. For the sample calculation, the GPower^®^ software version 3.1 was considered.

Statistical analyses were carried out using Minitab^®^ version 19, Jamovi^®^ version 2.4 software and Stata^®^ version 16.0. To determine the association between seasonality (dry season and rainy season) and the presence of Rotavirus, chi-square, Fisher’s Exact and Odds Ratio tests were applied. The associations were also estimated considering the type of source where the samples were collected. A normality analysis was performed using the Shapiro–Wilk test and central tendencies. Mann–Whitney and Kruskal–Wallis non-parametric tests were subsequently applied to compare concentrations of genomic copies, considering seasonality as a determining factor. A significance limit of 5% was considered to accept the hypotheses of associations and differences.

## 3. Results

**Figure 1:**
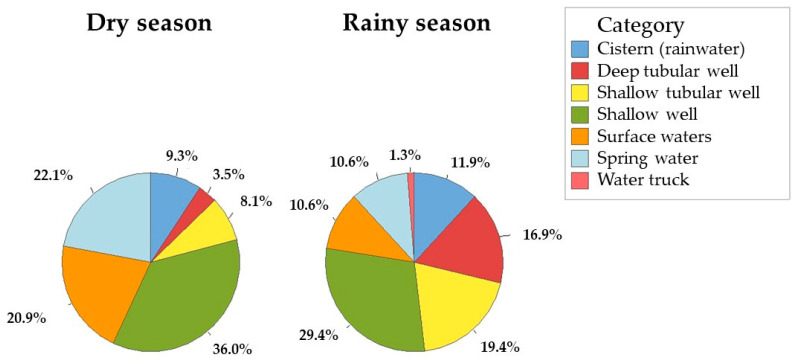
A presentation of the percentage of types of water sources where the research samples were collected. Each pie chart shows the percentages for each period, the dry period and the rainy period. Dry season (*n* = 86); rainy season (*n* = 160).

The frequencies of samples collected by the type of source (Figure 1), PCR and season can be seen in Table 3. The inferential analysis determined a strong association between the dry season and the presence of Rotavirus. When considering the general picture regardless of the type of source, the association test (χ^2^) resulted in 30.81; *p*-value < 0.001, a result similar to the Fisher’s Exact test with *p*-value < 0.001. The proportion of positive samples in the dry period, considering the type of source, resulted in being higher in shallow wells (*p*-value < 0.001), higher in surface waters (*p* = 0.001) and higher in spring water (*p*-value = 0.02). The other types of sources did not show significant proportional differences (Table 3).

Estimates of associations to determine the chances of positive samples according to seasonality were carried out using an Odds Ratio (OR) test (Table 4). In all cases, regardless of the type of source, higher frequencies of positive samples were observed in the dry season.

The overall picture presented OR = 4.82 [2.71–8.56; *p* < 0.0001)], that is, the chances of positive samples in the dry season are approximately five times higher when compared to the rainy season, an increase of 382% compared to the rainy season. The shallow wells also presented a higher percentage of positive samples in the dry season, OR = 10.63 [3.63–30.87; *p* < 0.0001]. Similarly, spring water showed a higher percentage of positive samples in the dry season, OR = 9.33 [1.01–86.36; *p* = 0.05] (Table 4). Only one surface water sample was positive in the dry season and there were no positive samples for truck water, which is why it was not possible to estimate the Odds Ratio (Table 4).

## 4. Discussion

Large numbers of pathogens such as viruses, bacteria and other microorganisms can be introduced to water sources due to the inefficiency or lack of protection of these sources. This makes the water quality unsuitable for consumption and causes outbreaks of waterborne diseases [19]. Isolated in groundwater, river water, sewage water and drinking water and causative of severe gastroenteritis in children, RV is responsible for approximately 24 million patients attending hospitals, 2.4 million hospitalizations, 114 million episodes of gastroenteritis and 450,000 deaths of children under 5 years per year, documented majority in low-income countries [20].

In the midwestern region of Brazil, where the state of Goiás is located, the highest prevalence of RV circulation occurs in the coldest months or in the dry period between May and September [21]. The RV-A found in the study samples was found in water samples both in the dry (54.7%) and rainy seasons (20%), but there was a higher prevalence and a strong statistical association between the dry season and the presence of RV (the association test (χ^2^) resulted in *p*-value < 0.001 and the Exact Test Fisher’s test with *p*-value < 0.001). The study shows that the chances of finding positive samples for RV in the dry season are five times greater than in the rainy season (OR = 4.82 [2.71–8.56; *p* < 0.0001)]). These results are in line with what was found in the study by Stobnicka-Kupiec and Górny [22] where the prevalence of positive RV samples found in winter (dry period) was higher than those found in summer (rainy period) (73.3% vs. 26.7%), with a statistics association between the prevalence and the period found (χ^2^: *p* = 0.028; Fisher’s Exact test: *p* = 0.033).

Outbreaks of infection not reported in the study areas, the elimination of feces with high concentrations of the virus (which can reach 10^8^ to 10^11^ particles/gram of feces) by infected patients, in addition to characteristics specific to the virus, may explain the greater detection of RV in the samples [12,23,24,25]. RVs are resistant to changes in pH, being inactivated only at a pH of 11.5, in addition to maintaining stability at relatively low humidity [25]. Its double-stranded RNA is surrounded by a capsid with a triple layer of protein, characteristics that give the virus greater resistance to endonuclease activities and protection against radiation through UV light [12,23,24,26].

In the dry months (winter) the UV irradiation is less intense (and therefore cannot penetrate cellular structures, damage the genetic material and interfere with viral reproduction) [23,27] and the temperatures are lower, which will lead to greater stability of Rotaviruses in the water. Viral capsid, nucleic acids and enzymes responsible for virus replication are damaged when present at high temperatures, preventing the adsorption of the virus to its host [23]. In tropical regions, RVs associated with the incidence of a diarrheal disease decrease of 10% for every 1 °C increase in temperature [28]. Also, it is known that in the colder and drier months, there is an immunological impact on the population, increasing susceptibility to these diseases [29].

In rural areas, not all homes are connected to the sewage collection network, but rather to septic tanks, from where sewage can drain into the ground and migrate to deeper layers, reaching groundwater [30,31,32]. Due to their extremely small size—between 25 and 100 nm—it allows the RVs to easily infiltrate and pass through soil pores, reaching these sources [30]. In addition, protection against UV radiation, low microbial activity and lower temperatures are reasons why viruses survive longer in groundwater [23]. It has been shown that RVs were able to survive for up to 7 months when stored in the dark [26]. For Fongaro et al. [27], in groundwater sources, the prevalence of RVs was higher in the rainy season than in the dry season (80% vs. 60%), and in our study, the presence of RV-A was positively correlated with dry and rainy periods (in shallow wells (*p*-value < 0.001) and spring water (*p*-value = 0.02)).

Shallow wells were the only groundwater source where RV contamination showed a higher percentage of positive samples in the dry season (OR = 10.63 [3.63–30.87; *p* < 0.0001]), with a strong statistical association (*p*-value < 0.001). Shallow wells are more vulnerable to viral contamination as the depth of the wells is generally smaller and may not have lining or protection factors, thus resulting in pipe failures and/or septic tank overflows, in addition to the presence of animals nearby and surface contaminants that can be taken to the wells through precipitation, increasing the contamination of this source by viruses [33,34].

Another groundwater source that also had a strong statistical association with the presence of RV was water springs (*p*-value = 0.02). The positive samples for adenovirus and rotavirus in a spring, found by Gonella et al. [35], are explained by the presence of animals in its surroundings, which may be transmitting these agents as it is located in an ecological park. Springs that are located in lower regions may be more subject to contamination than in higher regions; human density and agricultural activities are also factors that can lead to contamination from this source [36].

The discharge of sewage into surface water sources is a large source of fecal pathogens, as bodies of water that receive untreated sewage or are subject to inefficient treatment often constitute public water supply sources, making them a risk to those who use them [37,38]. In rural areas, most of the final disposal of sewage can occur directly on the ground or in streams, rivers and lakes, contaminating bodies of water and increasing the risk of waterborne diseases [39,40]. Collections carried out by Bortagaray et al. [28] in two rivers, Santa Lucia and Uruguay, obtained a statistically significant association with the presence of RV in the dry period (winter) (Uruguay river: *p* = 0.0014, Santa Lucia river: *p* = 0.0008). Similar to what was found by Bortagaray et al. [28], the present study only found the presence of RV in the dry period (colder months), with a strong statistical association between the viruses and the dry period (*p* = 0.002).

Temperature, the presence of microbiota and desiccation are factors that effectively contribute to the destruction and decomposition of viruses and microbial pathogens in surface water; in addition, when exposed to solar radiation (UV light), the viruses are readily inactivated [41,42]. The presence of native aquatic microorganisms, such as bacteria, would lead to bacterial production of proteolytic enzymes, which works as a mechanism for the antiviral activity of bacteria [6,43,44,45]. These factors may justify the absence of RV in surface samples during the rainy season, as the temperature is higher during this period.

## 5. Conclusions

The presence of pathogenic microorganisms in water is a public risk problem, enabling the emergence of outbreaks, endemics and epidemics. Describing or identifying the seasonal behavior of pathogens is important for prevention and attention to water treatment. In the present research, there was an association between the presence of Rotavirus and the dry period of the year when compared to the rainy period. It is possible to affirm that the dry period is associated with the presence of RV in surface and groundwater sources.

## Figures and Tables

**Table 1:** Location and coordinates of the municipalities and communities where the collections were carried out.

Municipalities (Community)	Latitude	Longitude	Water Samples/Site	Season
Simolândia (Castelo, Retiro e Três rios)	14°28′18″ S	46°29′11″ W	5	Dry and Rainy season
Nova Roma (Magalhães)	13°44′25″ S	46°52′52″ W	1	Dry season
Mineiros (Cedro)	17°34′43″ S	52°32′33′′ W	7	Dry season
Mimoso de Goiás (Queixo Dantas)	15°3′29″ S	48°9′33″ W	2	Dry season
Cavalcante (São Domingos)	13°47′51″ S	47°27′20″ W	3	Dry and Rainy season
Flores de Goiás (Canabrava)	14°26′18″ S	47°2′55″ W	14	Dry and Rainy season
Monte Alegre de Goiás (Pelotas)	13°25′14.16″ S	47°9′46.08″ W	14	Dry and Rainy season
Cidade Ocidental (Mesquita)	16°6′19″ S	47°57′0″ W	4	Dry season
São João d’Aliança (Forte)	14°42′31″ S	47°31′17″ W	2	Dry season
Niquelândia (Rafael Machado)	14°27′28″ S	48°27′59″ W	2	Dry season
Colinas do sul (José de Coleto)	14°8′47″ S	48°4′19″ W	1	Dry season
Campos Belos (Taquarussu)	13°1′31″ S	46°45′54″ W	13	Dry and Rainy season
Iaciara (Extrema)	14°5′45″ S	46°37′55″ W	19	Dry and Rainy season
Padre Bernardo (Sumidouro)	15°9′36″ S	48°17′2″ W	25	Dry and Rainy season
São Miguel do Araguaia (Lageado settlements)	13°16′30″ S	50°9′46″ W	27	Dry and Rainy season
Silvânia (São Sebastião da Garganta settlements)	16°39′32″ S	48°36′28″ W	27	Dry and Rainy season
Mineiros (Pouso Alegre)	17°34′08″ S	52°33′03″ W	1	Rainy season
Uruaçu (São Lourenço)	14°31′30″ S	49°08′27″ W	11	Rainy season
Goianésia (Itajá ii)	15°19′1″ S	49°7′1″ W	10	Rainy season
Professor Jamil (Rochedo)	17°15′03″ S	49°14′38″ W	20	Rainy season
Silvânia (João de Deus)	16°39′32″ S	48°36′28″ W	5	Rainy season
Nova Crixás (Landi)	14°05′52″ S	50°20′17″ W	8	Rainy season
Água limpa (Arraial da ponte)	18°04′26″ S	48°45′43″ W	1	Rainy season
Goiandira (Povoado Veríssimo)	18°07′58″ S	48°05′06″ W	1	Rainy season
Gameleira de Goiás (Olhos d’Agua)	16°27′50″ S	48°40′12″ W	4	Rainy season
Barro alto (Santo Antônio da Laguna)	14°58′15″ S	48°54′57″ W	9	Rainy season
Niquelândia (Povoado Vermelho)	14°28′26″ S	48°27′36″ W	2	Rainy season
Divinópolis de Goiás (Vazante)	13°17′42″ S	46°23′34″ W	2	Rainy season
Alto paraíso de Goiás (Povoado Moinho)	14°07′58″ S	47°30′36″ W	4	Rainy season
Posse (Baco Pari)	14°05′34″ S	46°22′08″ W	2	Rainy season
Total			246	

Note: west (W), south (S).

**Table 2:** Description of the RV primer used in the study.

Virus	Target Gene	Name	Sequence	Polarity	Position	Ta * (°C)	Amplicon (bp)
GARV	VP6	ROTAFEEVALE-FW	5′-GATGTCCTGTACTCCTTGT-3′	Sense	7–25 ^a^	60°	160
		ROTAFEEVALE-REV	5′-GGTAGATTACCAATTCCTCC-3′	Reverse	148–167 ^a^		

^a^ Sequence of primers described by Vecchia et al. * Annealing temperature.

**Table 3:** Association between the presence of Rotavirus and the period analyzed (dry season x rainy season), considering the font type as a dependent factor. All sources were considered in the analyses. Specific analyses were carried out for each font and general analysis.

Season	Font Type	Count	Positive	χ^2^ (*p*-Value)	Fisher’s *p*-Value
Dry season	Cistern (rainwater)	8 (9.3%)	4	3.43 (0.064)	0.145
Rainy season	Cistern (rainwater)	19 (11.9%)	3		
Dry season	Deep tubular well	3 (3.5%)	1	0.017 (0.894)	1
Rainy season	Deep tubular well	27 (16.9%)	3		
Dry season	Shallow tubular well	7 (8.1%)	4	(1.52) 0.218	0.387
Rainy season	Shallow tubular well	31 (19.4%)	10		
Dry season	Shallow well	31 (36%)	23	(21.43) < 0.001	<0.001
Rainy season	Shallow well	47 (29.4%)	10		
Dry season	Surface waters	18 (20.9%)	8	(10.28) 0.002	0.003
Rainy season	Surface waters	17 (10.6%)	0		
Dry season	Spring water	19 (22.1%)	7	(4.97) 0.026	0.044
Rainy season	Spring water	17 (10.6%)	1		
Rainy season	Water truck	2 (1.3%)	0	*No estimate*	1
Dry season	Total	86 (100%)	47	(30.81) < 0.001	<0.001
Rainy season	Total	160 (100%)	32		

**Table 4:** The association between seasonality and the presence of Rotavirus according to the OR test.

Font Type	OR	Lower	Upper	*p*-Value
Cistern (rainwater)	5.33	0.83	34.09	*0.17*
Deep tubular well	1.18	0.09	15.03	*0.59*
Shallow tubular well	2.80	0.52	14.95	*0.42*
Shallow well	10.63	3.63	30.87	*<0.0001*
Surface waters	-	-	-	*-*
Water truck	-	-	-	*-*
Spring	9.33	1.01	86.36	*0.05*
Total	4.82	2.71	8.56	*<0.0001*

## Data Availability

Data is contained within the article.
